# ﻿*Iguanainsularis* (Iguanidae) from the southern Lesser Antilles: An endemic lineage endangered by hybridization

**DOI:** 10.3897/zookeys.1086.76079

**Published:** 2022-02-17

**Authors:** Michel Breuil, David Schikorski, Barbara Vuillaume, Ulrike Krauss, Jennifer C. Daltry, Glenroy Gaymes, Joanne Gaymes, Olivier Lepais, Nicolas Bech, Mišel Jelić, Thomas Becking, Frédéric Grandjean

**Affiliations:** 1 Muséum national d’Histoire naturelle, Laboratoire des Reptiles et Amphibiens, Bâtiment 30, 57, rue Cuvier, CP n° 30, 75231 Paris cedex 05, France Muséum national d’Histoire naturelle Paris France; 2 Laboratoire Labofarm-Genindexe, 4 rue Théodore Botrel, 22600 Loudéac, France Laboratoire Labofarm-Genindexe Loudéac France; 3 Maison du Soleil, Dauphin Road, La Borne, PO Box GM 1109, Saint Lucia, West Indies Unaffiliated La Borne Saint Lucia; 4 Fauna & Flora International, David Attenborough Building, Pembroke Street, Cambridge CB2 3QZ, UK Fauna & Flora International Cambridge United Kingdom; 5 Re:wild, PO Box 129, Austin, TX 78767, USA Re:wild Austin United States of America; 6 Science Initiative for Environmental Conservation and Education, Kingstown, St Vincent & the Grenadines Science Initiative for Environmental Conservation and Education Kingstown Saint Vincent and The Grenadines; 7 INRAE, Univ. Bordeaux, BIOGECO, 69 route d’Arcachon, 33612 Cestas Cedex, France Univ. Bordeaux Bordeaux France; 8 Laboratoire Écologie et Biologie des Interactions, équipe EES, UMR CNRS 7267, Université de Poitiers, 5 rue Albert Turpin, 86073 Poitiers Cedex 9, France Université de Poitiers Poitiers France; 9 Department of Natural Sciences, Varaždin City Museum, Šetalište Josipa Jurja Strossmayera 3, 42000 Varaždin, Croatia Varaždin City Museum Varaždin Croatia

**Keywords:** Caribbean, *Iguanainsularisinsularis*, *Iguanainsularissanctaluciae*, introgression, invasive alien species, microsatellites, ND4, PAC

## Abstract

The newly described horned iguana*Iguanainsularis* from the southern Lesser Antilles is separated in two easily recognized subspecies: *I.insularissanctaluciae* from St. Lucia and *I.insularisinsularis* from the Grenadines. Its former description is completed by the use of 38 new samples for genetic and morphological analysis. Seventeen microsatellites were used to estimate genetic diversity, population structure and the level of introgression with other *Iguana* species over nearly the whole range of the species. ND4 and PAC sequences were also used to better characterize hybridization and to complete the description of this lineage. The *I.insularis* population of St. Vincent shows a high level of introgression from *I.iguana* whereas in the Grenadines, most islands present pure *insularis* populations but several show evidence of introgressions. Of the two remaining populations of *I.insularissanctaluciae*, only one is still purebred. The recent identification of this and other distinct insular species and subspecies in the eastern Caribbean, and evaluation of where hybridization has occurred, are timely and important because the native iguanas are in urgent need of conservation action. Among the greatest threats is the ongoing human-mediated spread of invasive iguanas from Central and South America, which are destroying the endemic insular lineages through multiple diachronic introgression events.

## ﻿Introduction

Resolving taxonomic or phylogenetic uncertainties and delineating management units according to the genetic characteristics of populations are important in conservation biology (Groom et al. 2006; Pasachnik et al. 2009, 2020; Malone et al. 2017). This is particularly challenging when reproductive barriers are absent between native and alien species and interspecific hybridization occurs; sometimes leading to the extinction of rare taxa through genetic swamping. A good example concerns the genus *Iguana* which was long thought to be represented by only two species (Lazell 1973): the Lesser Antillean iguana *I.delicatissima* (Laurenti 1768), endemic to the Lesser Antilles, and the invasive common or green iguana (*Iguanaiguana*) [Linnaeus 1758], with a large distribution range encompassing Central America and some offshore islands, the north of South America and offshore islands (e.g., the ABC Islands, Los Roques and Margarita). Recently, Vuillaume et al. (2015) reported that *I.delicatissima* hybridizes with invasive *I.iguana* in the Lesser Antilles, resulting in the progressive elimination of *I.delicatissima* by genetic swamping.

The range of the *Iguanaiguana* complex sensu van den Burg et al. (2021) covers approximately 5 million km^2^ (Breuil 2013) and contains independent lineages identified by Stephen et al. (2013). Thus, obtaining sufficient numbers of individuals over the entire geographic range for comprehensive phylogenetic and taxonomic studies covering the entire range is difficult. However, Buckley et al. (2016) acknowledged that Breuil (2013) found significant morphological differences between the Saba, St. Lucia, and South American populations. Taxonomic interpretation across the global range may be much more complicated than the conclusions drawn from our Lesser Antilles samples (Breuil et al. 2019, 2020) as suggested by recent research on the ABC Islands and Colombia (van den Burg and Malone 2018). In addition, numerous iguana translocations have occurred in the Lesser Antilles since the Caribbean period (Bochaton et al. 2015; Vuillaume et al. 2015; De Jésus Villanueva et al. 2021) and have altered the original endemic populations.

The iguanas used by Breuil et al. (2019, 2020) to differentiate Lesser Antillean taxa from continental iguanas originate from northern South America (French Guiana), representing only 1% of the global range. Furthermore, van den Burg et al. (2021) showed that these French Guiana iguanas do belong to the same genetic group as those from Surinam, Trinidad, Venezuela (Bolivar Rio Caroni) and Brazil (Alter do Chao), a conclusion previously reached by Stephen et al. (2013) using three nuclear genes and one mitochondrial gene. We therefore considered that these French Guiana iguanas correspond to the species *Iguanaiguana* (Breuil 2013, 2016; Breuil et al. 2019, 2020) described by Linnaeus (1758) based on the type locality assigned to this species by Hoogmoed (1973) “confluence of the Cottica River and Perica Creek, Surinam” and Duellman (2012) “vicinity of Paramaribo, Surinam”. Thus, the common iguanas of northern South America do belong to the species *Iguanaiguana* described by Linnaeus without prejudging the taxonomic status of populations in the rest of South America. This is also the position taken by Buckley et al. (2016) if it is considered that those from Central America belong to the species *Iguanarhinolopha* (Wiegemann 1834).

**Figure 1.**
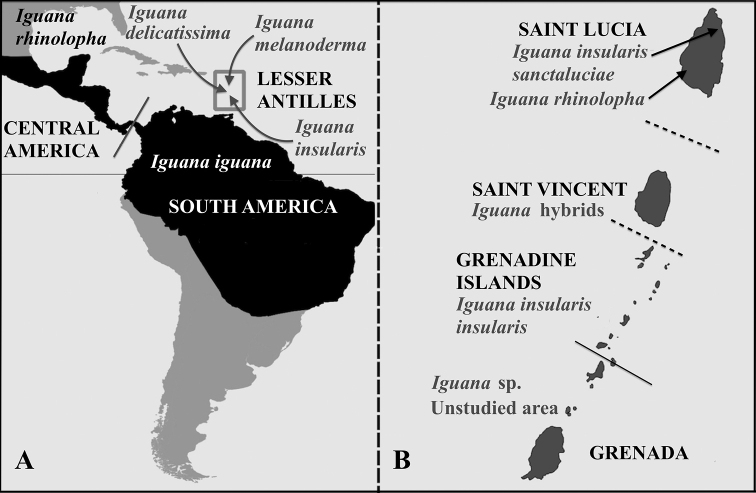
**A** distribution of the five *Iguana* species recognized by Breuil et al. (2020). The Lesser Antilles contain three endemic species (*I.delicatissima, I.insularis, I.melanoderma*) and the invasive aliens *I.iguana* and *I.rhinolopha*, which hybridize with the endemic insular taxa. The grey line between Central and South America indicates the approximate limit between the two recognized continental species, but the taxonomy and distribution of iguanas in this area warrants further investigation **B** the three geological banks referred to in this paper are separated by two horizontal dash black lines. The black line indicates the political boundary between the country of St. Vincent and the Grenadines to the north and Grenada to the south. The iguanas are named according to the results of this study and Breuil et al. (2020).

Based on both genetic and morphological data, five species are now recognized (Breuil 2013, 2016; Breuil et al. 2020; Breuil 2021; van den Burg et al. 2021; Caribherp 2021) without considering some regions for which we had no data for these studies (east Ecuador and Columbia, NW Venezuela and ABC Islands, South Brazil): *I.iguana* endemic to north South America, east of the Andes, *I.rhinolopha* endemic to Central America, and, in the eastern Caribbean, *I.delicatissima* in the northern Lesser Antilles, *I.melanoderma* endemic to Saba and Montserrat, and *I.insularis* (Breuil et al. 2020) endemic to the southern Lesser Antilles (Fig. 1). Van den Burg and Malone (2018) argued for a revision of the taxonomy of the *Iguanaiguana* complex and our proposals are in total accordance with published data. However, the Reptile Database (2021) prefers to consider *insularis*, *sanctaluciae* and *melanoderma* as subspecies of *Iguanaiguana* and follows the opinion of Lazell (1973) by not recognizing *rhinolopha* as a subspecies of *Iguanaiguana* nor as a species on a morphological basis. This work was impacted by the low number of samples, their low geographic coverage and the long overlooked hybridization between *Iguanaiguana* and *Iguanadelicatissima* that has blurred the morphological distinctions between the different lineages. Given that *I.delicatissima* and *I.iguana* readily interbreed and produce fertile hybrids, interspecific hybridizations could be widespread in the genus *Iguana.* To inform conservation management, it is important identify which populations of endemic lineages are still purebred and which show evidence of hybridization. This can be done only if the different lineages are well characterized by means of genetic and morphological data.

The newly described horned insular iguana*Iguanainsularis* from the southern Lesser Antilles is separated in two subspecies, *I.insularissanctaluciae* from St. Lucia and *I.insularisinsularis* from the Grenadines. The first descriptions of these taxa were supported by, morphology, including scales and color, and genetic criteria (Breuil et al. 2019). For example, the dewlap of adult iguanas from St. Lucia (*I.insularissanctaluciae*) becomes totally black with age and the body barred with broad black bands, whereas the dewlap of the Grenadines pink rhino iguana (*I.insularisinsularis*) is typically light cream to cream, its bands are narrower, and, in old individuals, markings fade until the animal is almost uniform light cream to nearly white. The genetic analysis was based on a relatively large number of individuals from St. Lucia but only four individuals from two islands in the Grenadines (Breuil et al. 2019) and none from St. Vincent. This limited genetic sampling made it difficult to draw firm conclusions about the distribution of *I.insularisinsularis* and its relationship to *sanctaluciae*.

The present paper adds distribution and genetic data from a further 34 individuals sampled from 20 Grenadine Islands and, for the first time, four specimens from St. Vincent. Seventeen microsatellites were used to estimate genetic diversity, population structure and differentiation between the two subspecies as well as the level of introgression with other *Iguana* species. In addition, both the mitochondrial ND4 and nuclear gene PAC sequences were obtained to gain valuable additional information on genetic variation and hybridization within *Iguanainsularis*. This work aims to inform conservation strategies to preserve the genetic integrity of purebred populations of both subspecies, *I.insularisinsularis* and *I.insularissanctaluciae*.

## ﻿Materials and methods

### ﻿Field methods

**Figure 2.**
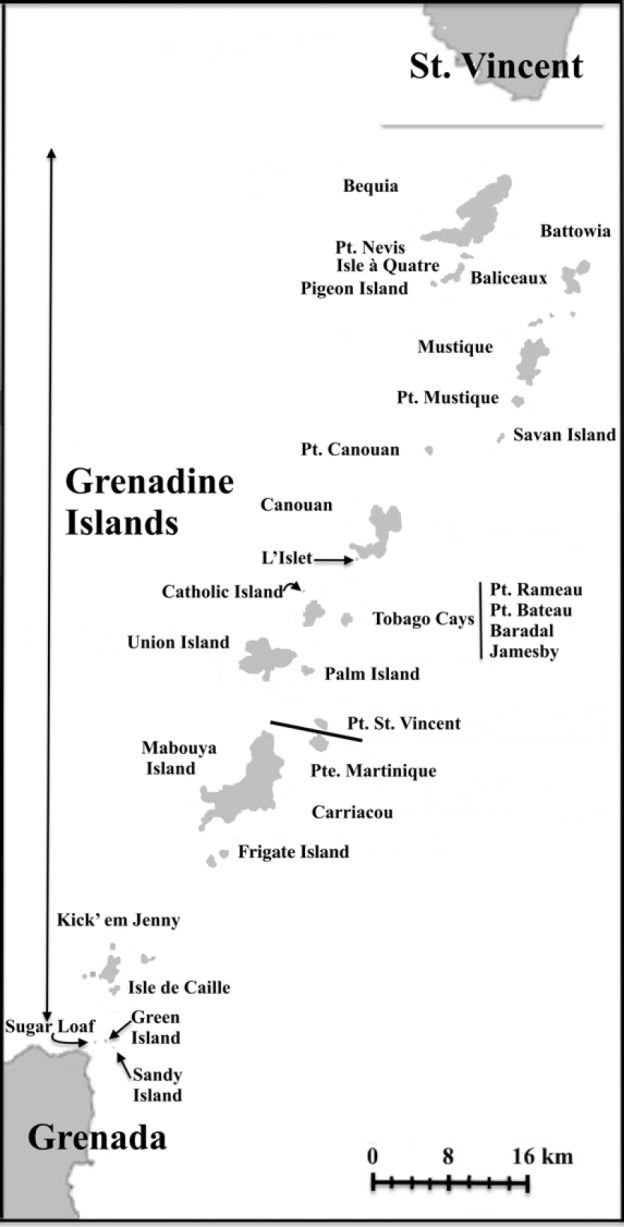
Distribution of iguanas in the Grenadine Islands (modified from Henderson and Powell 2018; Breuil et al. 2019). Note there are alien iguanas on some islands and not all of the island clusters shown here have purebred populations of *I.insularisinsularis*. The grey line south of St. Vincent marks the break between the Saint Vincent Bank to the north and the Grenada Bank to the south. The black line between Petit St. Vincent and Petit(e) Martinique shows the political boundary between St. Vincent and the Grenadines to the north and Grenada to the south. The Grenadine Islands form an archipelago from the south of St. Vincent to the north of Grenada. Almost all the Grenadine Islands in St. Vincent and the Grenadines were surveyed during this work and, with the exception of Savan Island, had iguanas at the time of our visits. Petit Mustique was inaccessible during our survey. The islets of Sugar Loaf, Green Island,c and Sandy Island (all in Grenada) are reported to no longer have iguanas (Henderson and Powell 2018) but iguanas fitting the morphology of *I.insularisinsularis* have been observed on Carriacou and Mabouya Islands (see text).

A total of 24 islands and islets were surveyed in St. Vincent and the Grenadines, of which 19 were confirmed to have iguanas. In the Grenadines, the islands surveyed by JD, GG, JG, and colleagues were Union, Tobago Cays, Petit St. Vincent, Canouan and adjacent islands from 5–8 August 2018; Bequia, Battowia Group and adjacent islands from 15–16 August 2018; Bequia alone on 30 August 2018; and Mustique and its adjacent islands on 20 and 21 August 2018. Petit Canouan, Petit Nevis, Isle à Quatre, Pigeon (Ramier) and Mustique were visited between 10–15 September 2019 (Fig. 2). No surveys were conducted on Grenadines islands within Grenada’s borders. St. Vincent was also visited by GG and JG, but sampling was confined to Kingstown Botanical Garden.

The iguanas were captured by hand or with a noose. Measurements (snout-vent length and total length) and photographs were taken. Tissue samples (tail tip or shed skin) were collected and preserved in 70% ethanol. The procedure was done as quickly as possible, and the iguanas were released back in their habitat. Photographs were also taken of individuals that evaded capture. Iguanas observed and/or caught in St. Vincent and the Grenadines were identified by using the suite of morphological traits recognized as diagnostic by Breuil (2013, 2016) and Breuil et al. (2019, 2020).

### ﻿Genetic samples and genetic diversity: microsatellites

For this genetic study, we took biopsies from 34 iguanas from 15 Grenadine Islands and four iguanas from St. Vincent. These 38 samples were genotyped using 17 microsatellite markers amplified as described by Valette et al. (2013) and Vuillaume et al. (2015) (whereas only 15 were used in Breuil et al. 2019) (Tables 1, 2). Subsequently, amplification products were resolved by electrophoresis on an ABI PRISM 3130 Genetic Analyzer. Product sizes were determined using the GeneMapper software (Applied Biosystems, Saint Aubin, France), followed by verification by eye.

These individuals were considered to belong to one group according to the description of Lazell (1973) (St. Vincent and the Grenadines) and compared to other groups identified by Breuil et al. (2019, 2020) (Table 1). We tested all these groups of individuals for departures from Hardy-Weinberg expectations using the software GenAlEx (Peakall and Smouse 2006). Linkage disequilibrium was assessed for each specific microsatellite marker as implemented in FSTAT ver. 2.9.3.2 (Goudet 2001) (with 1,200 permutations). We adjusted the levels of significance for multiple tests using the standard Bonferroni correction (Rice 1989). Further, we assessed genetic polymorphism with the Allelic richness (*Ar*), expected heterozygosity (*He*) and *Fis* using FSTAT ver. 2.9.3.2 (Goudet 2001) with 1,200 permutations.

**Table 1.** Iguanas sampled for genetic analysis.

Localities	Taxa	Sample size
French Guiana	*iguana*	7
St. Lucia (South West)	*rhinolopha*	7
St. Lucia (Grand Anse)	hybrid^#^	4
St. Lucia (Louvet)	*sanctaluciae*	13
St. Vincent (Kingstown)	hybrid^#^	4
Grenadines (Battowia)	*insularis*	3*
Grenadines (Balliceaux)	*insularis*	1*
Grenadines (Petit Nevis)	*insularis*	1*
Grenadines (Pigeon)	*insularis*	2*
Grenadines (Mustique)	*insularis*	4*
Grenadines (Petit Canouan)	*insularis*	3*
Grenadines (Canouan)	*insularis*	1*
Grenadines (L'Islet)	*insularis*	2*
Grenadines (Tobago Cays: Baradal)	*insularis*	4*
Grenadines (Tobago Cays: Jamesby)	*insularis*	4*
Grenadines (Tobago Cays: Petit Bateau)	*insularis*	2*
Grenadines (Tobago Cays: Petit Rameau)	*insularis*	2*
Grenadines (Union Island)	*insularis*	(3* + 1)
Grenadines (Palm Island)	*insularis*	3
Grenadines (Petit St. Vincent)	*insularis*	2*
Montserrat	*melanoderma*	11
Saba	*melanoderma*	6

* denotes individuals that were new to this study and not presented in previous publications. ^#^ hybrid indicates introgressed populations that were identified by a previous study for St. Lucia (Breuil et al. 2019) and by morphology and genetic analysis for St. Vincent.

### ﻿Genetic structure and introgression

For these analyses, we used microsatellite data from the first four iguanas captured in the Grenadines (*I.insularisinsularis* from Union and Palm Islands) in 2018, 17 iguanas from northeast St. Lucia corresponding to *I.insularissanctaluciae*, seven *I.rhinolopha* collected from southwest St. Lucia (where this species is an invasive alien), seven *I.iguana* from French Guiana (see Breuil et al. 2019) and 17 *I.melanoderma* from Saba and Montserrat (Breuil et al. 2020) to obtain information about introgression and genetic structure (Table 1). All these analyses were made using 17 microsatellite markers (Table 2).

**Table 2.** Summary of the genetic diversity parameters for each locus and each locality.

Loci	Parameters	Groups of individuals							
		*iguana* French Guiana	*rhinolopha* St. Lucia	Hybrid St. Lucia Grand Anse	*sanctaluciae* St. Lucia Louvet	*insularis* St. Vincent and the Grenadines	*melanoderma* (Montserrat)	*melanoderma* (Saba)	All
		*n* = 7	*n*= 7	*n* = 4	*n* = 13	*n* = 42	*n* = 11	*n* = 6	90
L2	***He***	0.262	0.476	0.583	0.000	0.587	0.245	0.000	0.320
	***Ar***	1.505	1.789	1.929	1.000	2.195	1.470	1.000	2.318
	***Fis***	-0.091	1.000	0.571	NA	0.716	-0.111	NA	0.428
L3	***He***	0	0.000	0.000	0.000	0.000	0.000	0.000	0.000
	***Ar***	1.000	1.000	1.000	1.000	1.000	1.000	1.000	1.29
	***Fis***	NA	NA	NA	NA	NA	NA	NA	NA
L5	***He***	0.524	0.690	0.750	0.000	0.000	0.364	0.333	0.380
	***Ar***	1.915	2.538	2.643	1.000	1.000	1.674	1.576	1.666
	***Fis***	-0.091	-0.448	0.000	NA	NA	-0.250	1.000	0.042
L6	***He***	0.524	0.429	0.583	0.000	0.260	0.564	0.2	0.366
	***Ar***	1.869	1.789	1.971	1.000	1.522	2.154	1.400	2.218
	***Fis***	0.727	-0.333	0.143	NA	0.634	0.355	0.000	0.254
L8	***He***	0.262	0.5	0	0	0	0	0	0.109
	***Ar***	1.505	2.000	1.000	1.000	1.000	1.000	1.000	1.117
	***Fis***	-0.091	0.000	NA	NA	NA	NA	NA	-0.046
L9	***He***	0.607	0.619	0.583	0.000	0.675	0.672	0.717	0.553
	***Ar***	2.326	2.181	1.971	1.000	2.478	2.507	2.461	2.652
	***Fis***	-0.412	0.308	0.143	NA	0.577	0.256	0.535	0.235
L13	***He***	0.000	0.000	0.583	0.000	0.266	0.000	0.000	0.121
	***Ar***	1.000	1.000	1.971	1.000	1.496	1.000	1.000	1.866
	***Fis***	NA	NA	0.143	NA	0.373	NA	NA	0.258
L14	***He***	0.143	0.000	0.250	0.091	0.157	0.467	0.683	0.256
	***Ar***	1.286	1.000	1.500	1.182	1.308	1.845	2.434	1.995
	***Fis***	0	NA	0	0	0.546	-0.5	0.024	0.012
L15	***He***	0.679	0.357	0.250	0.000	0.092	0.091	0.000	0.210
	***Ar***	2.426	1.670	1.500	1.000	1.180	1.182	1.000	1.806
	***Fis***	0.158	-0.200	0.000	NA	0.484	0.000	NA	0.088
L16	***He***	0.143	0.733	0.000	0.000	0.175	0.000	0.000	0.150
	***Ar***	1.286	2.461	1.000	1.000	1.335	1.000	1.000	1.452
	***Fis***	0.000	0.773	NA	NA	0.457	NA	NA	0.410
L17	***He***	0.488	0.548	0.750	0.000	0.184	0.000	0.000	0.281
	***Ar***	1.955	1.915	2.557	1.000	1.367	1.000	1.000	2.236
	***Fis***	0.415	0.478	0.333	NA	0.736	NA	NA	0.491
L18	***He***	0.533	0.381	0.000	0.000	0.177	0.403	0.000	0.213
	***Ar***	1.939	1.670	1.000	1.000	1.335	1.810	1	1.978
	***Fis***	-0.250	0.625	NA	NA	1.000	0.448	NA	0.456
L19	***He***	0.524	0.000	0.750	0.000	0.218	0.650	0.533	0.382
	***Ar***	1.930	1.000	2.643	1.000	1.428	2.381	1.919	2.172
	***Fis***	-0.364	NA	0.000	NA	0.344	-0.119	0.063	-0.015
L20	***He***	0.655	0.524	0.750	0.000	0.198	0.445	0.000	0.367
	***Ar***	2.411	1.915	2.557	1.000	1.383	1.809	1.000	2.422
	***Fis***	-0.091	-0.091	0.333	NA	0.759	-0.429	NA	0.096
L23	***He***	0.821	0.350	0.750	0.000	0.296	0.000	0.200	0.345
	***Ar***	2.921	1.667	2.643	1.000	1.616	1.000	1.400	2.454
	***Fis***	0.304	-0.143	0	NA	0.239	NA	0.000	0.080
L24	***He***	0.000	0.000	0.000	0.000	0.000	0.000	0.000	0.000
	***Ar***	1.000	1.000	1.000	1.000	1.000	1.000	1.000	1.000
	***Fis***	NA	NA	NA	NA	NA	NA	NA	NA
L25	***He***	0.000	0.000	0.750	0.520	0.000	0.000	0.303	0.315
	***Ar***	1.000	1.000	2.643	1.904	1.000	1.000	1.576	2.089
	***Fis***	NA	NA	0.000	-1.000	NA	NA	1.000	-0.152
All	***He***	0.363	0.330	0.431	0.036	0.193	0.229	0.174	0.257
	***Ar***	1.722	1.623	1.855	1.063	1.391	1.461	1.339	1.925
	***Fis***	0.038	0.198	0.118	-0.846	0.583	-0.017	0.432	0.288

N: number of analyzed samples; He: expected heterozygosity, *Ar*: allelic richness and *Fis* : inbreeding coefficient. In italics and bold: the *Fis* value with significant departures from Hardy-Weinberg expectations (i.e., significantly different from 0; P < 0.0005 after Bonferroni adjustment). NA = not available. The iguanas of St. Vincent were included in the group *insularis* according to their geographical origin.

We computed pairwise fixation index (*Fst*) values between groups of individuals (Weir and Cockerham 1984) using fstat v. 2.9.3.2 (Goudet 2001). We adjusted the levels of significance for multiple tests using the standard Bonferroni correction as implemented in fstat v. 2.9.3.2 (Goudet 2001).

We conducted a Discriminant Analysis of Principal Components (DAPC) in the *adegenet* package (Jombart 2008; Jombart et al. 2010) for R version 3.5.0 to investigate population genetic structure at the individual level. We first performed a Principal Component Analysis (PCA) to transform the raw genetic data retaining all principal components to maximize the variation of the original data. The best number of clusters K was estimated using the function *find.clusters* that implemented a K-means clustering minimizing the variation within clusters and a Bayesian Information Criterion (BIC) approach. We assumed a maximum number of 10 clusters and ran the K-means algorithm with 1,000 random starting values and 10^8^ iterations to ensure convergence. A Discriminant Analysis (DA) was then applied with the DAPC function using 30 principal components explaining more than 95% of the total variance of the data and retaining two discriminant functions that carried most information.

At the individual level, we also accessed the genetic structure using the Bayesian approach implemented by the software STRUCTURE (Pritchard et al. 2000). This clustering approach estimated both the number (*K*) of genetic cluster(s) and the admixture coefficient of individuals to be assigned to the inferred clusters. We selected the admixture model and the option of correlated allele frequencies among populations. As recommended by Evanno et al. (2005), we replicated 20 independent runs for each value of *K* (with *K* varying from 1 to 10) with a total of 100,000 burn-in and 100,000 recorded iterations. To determine the number of genetic clusters from structure analyses, we used the STRUCTURE HARVESTER program (Earl and VonHoldt, 2011) to compare the mean likelihood computed from the 20 independent runs.

The best number of clusters was determined using the hierarchical approach delta K method (Evanno et al. 2005). In a first stage, the uppermost hierarchical structure was determined by determining the best number of clusters using delta K on the entire dataset. In a second stage, independent analyses were performed with individual belonging to each genetic cluster identified in the first stage to identify more refined population genetic structure within main genetic clusters. The total number of clusters was then determined by summing up the number of clusters in analyses of the subset of the data. The final result was obtained by selecting the most likely run from the entire dataset analysis (i.e., showing the highest likelihood) within repeated runs at optimal K value. The R package ape (Paradis et al. 2019) was used to build a genetic distance tree based on the allele frequency divergence among genetic clusters computed by STRUCTURE.

### ﻿Mitochondrial and nuclear genes

ND4 sequences were obtained from 14 individuals from St. Vincent and the Grenadines following the methods of Breuil et al. (2019), and the PAC region was sequenced from five individuals from St. Lucia, three from St. Vincent, and 10 from the Grenadine Islands according to the protocol of Stephen et al. (2013). These were used to gain a more complete insight into introgression because these two genes are diagnostic of *Iguanainsularissanctaluciae* in St. Lucia (Stephen et al. 2013) and, as discovered in this work, also of *I.insularisinsularis* in St. Vincent and the Grenadines.

## ﻿Results

### ﻿Geographical distribution of iguanas in St. Vincent and the Grenadines

This analysis confirms the presence of Grenadines pink rhino iguanas (*I.insularisinsularis*) on the following Grenadine Islands (listed from north to south): Bequia, Petit Nevis, Isle à Quatre, Pigeon Island, Battowia, Baliceaux, Mustique, Petit Canouan, Canouan, L’Islet, Catholic Island, Mayreau, Baradal, Jamesby, Petit Bateau, Petit Rameau, Union Island, Palm Island, Frigate Rock, and Petit Saint Vincent (Fig. 2). No iguanas were observed on Petit Tabac, Church Cay, West Cay, the Pillories (small cays between Battowia and Baliceaux) or Savan Island. All the aforementioned islands are within the political boundary of St. Vincent and the Grenadines: no genetic data were obtained from islands belonging to Grenada.

**Figure 3.**
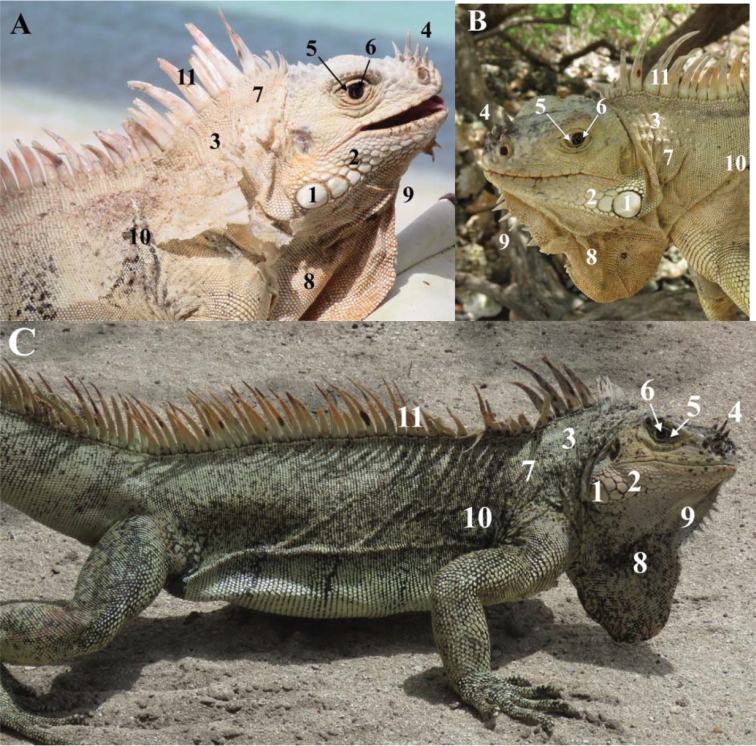
Photographs of three adult male *Iguanainsularisinsularis* on the Tobago Cays (Grenadine Islands). **A** IGU105 (Baradal). This male has the typical coloration of *I.insularisinsularis* with faint black banding, eye with visible white area, nasal horns, small subtympanic plate and very few and small tubercles on the neck, but also presents atypical sublabial scales and conical scales on the nape **B** IGU112 (Petit Rameau) is a typical older male *I.insularisinsularis*, with no black bands on the body (not shown in this photograph) **C** IGU110 (Petit Bateau) has a body with small and narrow ventral black bands and numerous black scales on the body. 1. relatively small subtympanic plate 2. mosaic of small scales 3. very low to low number of small neck tubercles 4. lateral and median horns 5. white visible in the eye. 6. brown eye 7. light cream coloration in old adults; green in juveniles and younger adults 8. light dewlap with some black scales (**C**) 9. small number of small gular spikes (not always visible on the photographs) 10. light body with different degrees of persistence of black stripes 11. light and high dorsal spikes with a pink or orange hue.

**Figure 4.**
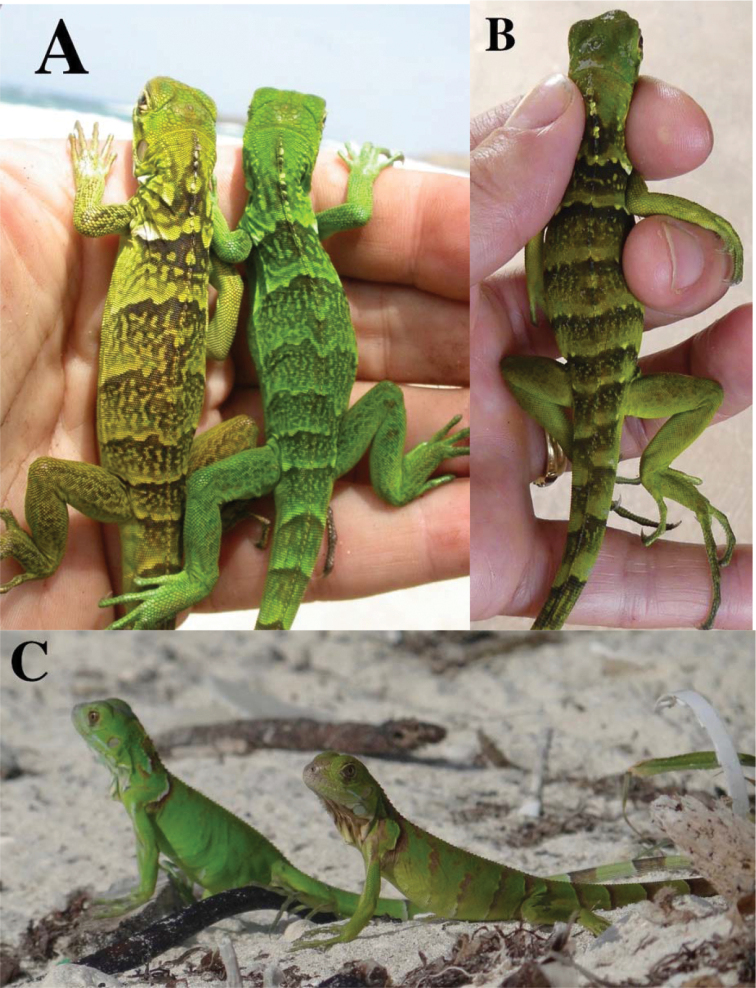
**A***Iguanainsularissanctaluciae* hatchlings (northeast Saint Lucia) **B** hatchling *I.insularisinsularis* (IGU143, Union Island, Grenadines). The *I.insularis* hatchlings in both **A** and **B** show strong dark green to light green banding on the body and the tail, with a white mark at the scapular level **C** hatchlings alien *I.rhinolopha* in Florida. These iguanas have a nearly uniform green body with only some brown narrow banding on the body.

All the iguanas captured and/or photographed from the Grenadines showed characteristics consistent with *I.insularisinsularis* (Breuil et al. 2019). For example, 12 iguanas from the Tobago Cays (Baradal, Jamesby, Petit Bateau, and Petit Rameau, Fig. 3) were adults that had lost their juvenile green coloration (Fig. 4), 11 of which had black stripes of variable intensity and width on a pale body, often with a pinkish hue (hence their trade name “zebra iguanas” or “Grenadines pink rhino iguanas”). Some iguanas had lost their black stripes, with only a few dark scales remaining. These iguanas were typically the largest and presumably oldest ones encountered. The largest individual (136 cm total length) was captured on Petit Bateau.

**Figure 5.**
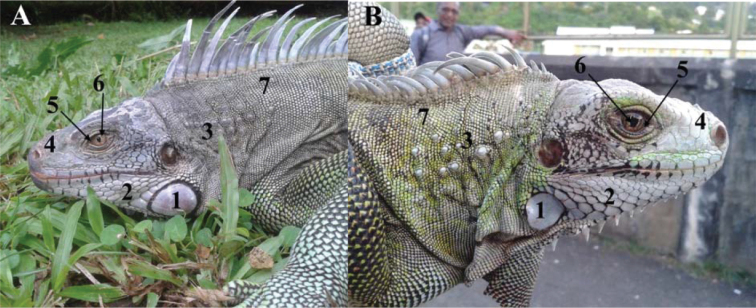
Hybrid iguanas IGU139 **A** and IGU141 **B** from the island of St. Vincent. (see text for more information) 1. medium (**B**) to large subtympanic plate (**A**) 2. mosaic of small scales 3. low number of small neck tubercles 4. no horn (**A**) or very short horns (**B**) 5. no white visible in the eye 6. yellowish-brown eye (**A**) or light brown eye (**B**) 7. grey-green coloration (**A, B**) or green-black coloration (**B**). None of these morphological features conform with the characteristics of *I.insularisinsularis*.

Three out of the four iguanas captured on St. Vincent (Kingstown Botanical Garden) were photographed (Fig. 5) and did not present any of the diagnostic characteristics of *I.insularis* described by Breuil et al. (2019). IGU139 (Fig. 5) is an old male with an elongated head, no nasal horns, a light eye with no white visible, a huge subtympanic plate, a mosaic of small scales anterior to this plate and a green body with light grey dorsal spines. This phenotype can be interpreted as an intermediate between *I.iguana* from French Guiana and *I.rhinolopha* with no apparent morphological traits of *I.insularis* apart from a low number of small to medium-sized tubercles on the neck. IGU140 was a young individual without horns, but other diagnostic characters could not be checked because of its age. IGU141 (Fig. 5) was a female that did not present any morphological characteristics of *I.insularis*. The very small nasal horns cannot be interpreted on this picture as typical of *rhinolopha* or *insularis* or intermediate between them. This individual possessed black scales between the eye and the tympanum that forms a kind of discontinuous spot (not dissimilar to the black spot of *I.melanoderma*: Breuil et al. 2020). Overall, these three iguanas had a phenotype most similar to *I.iguana* from French Guiana.

### ﻿Genetic diversity

No linkage disequilibrium was detected after applying a Bonferroni correction (p-value threshold after Bonferroni adjustment, *P* = 0.0005). Only eight of the 105 groups of individuals/locus combinations deviated significantly from Hardy-Weinberg expectations (adjusted p-value threshold after Bonferroni adjustment, *P* = 0.0004). These deviations occurred only for populations of *I.insularis* and likely resulted from a Walhund effect because the individuals came from different Grenadine islands and likely displayed different genetic signatures. All microsatellite loci were polymorphic with an allelic richness (*Ar*) ranging from 1 to 2.921 and a genetic diversity (*He*) ranging from 0 to 0.821 across groups of individuals (Table 2).

### ﻿Population structure

**Figure 6.**
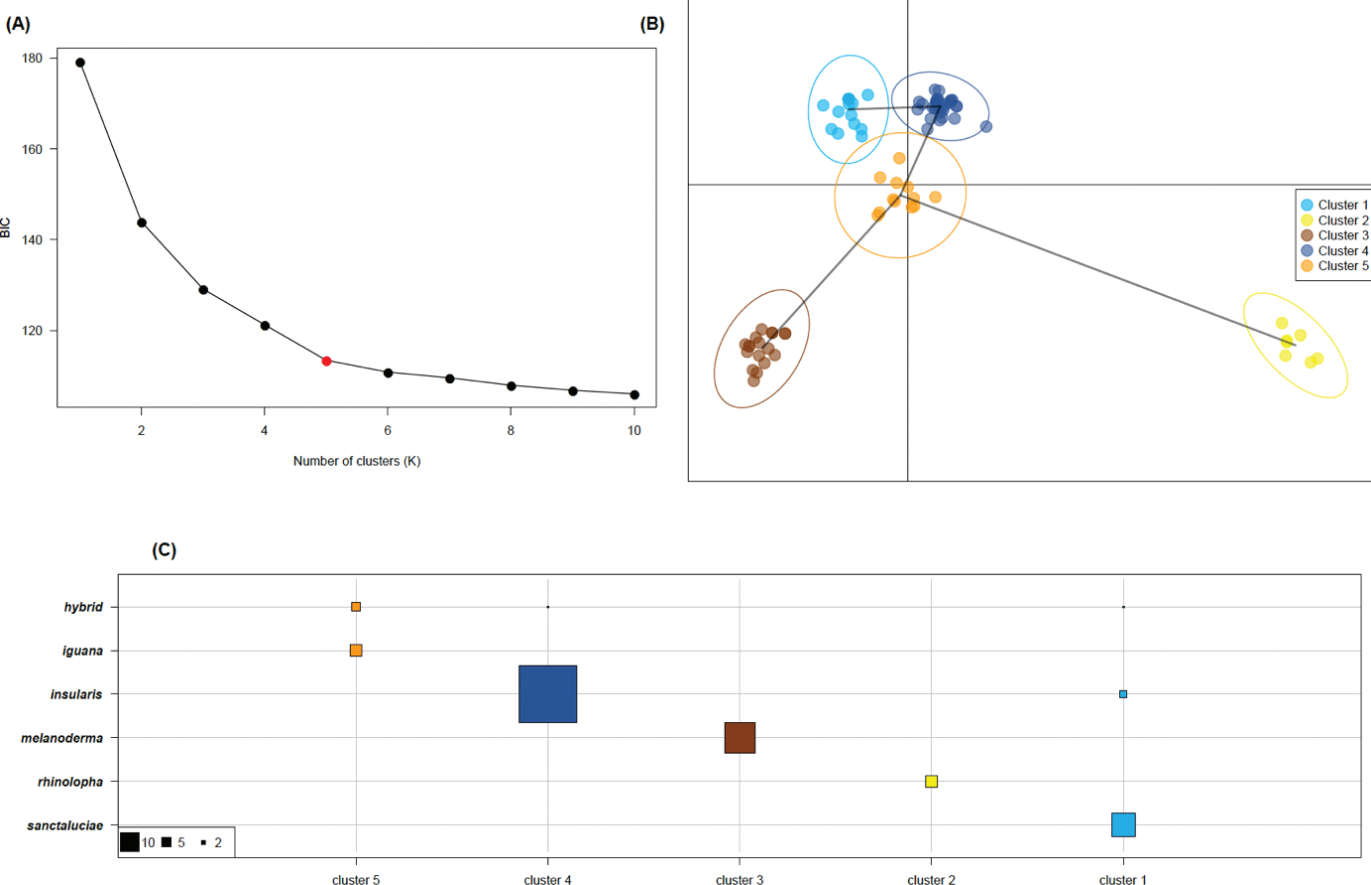
Discriminant Analysis of Principal Components (DAPC). **A** variation of the Bayesian Information Criterion (BIC) as a function of the assumed number of genetic clusters (K) **B** scatterplot representing individual (dots) and clusters (inertia ellipse) location in the principal component space **C** correspondence between species determination (in line) and genetic cluster (in column). The taxa names refer to species level for all them except for *insularis* and *sanctaluciae* which are the two subspecies of *Iguanainsularis*. Hybrid refers to the population of St. Vincent.

**Figure 7.**
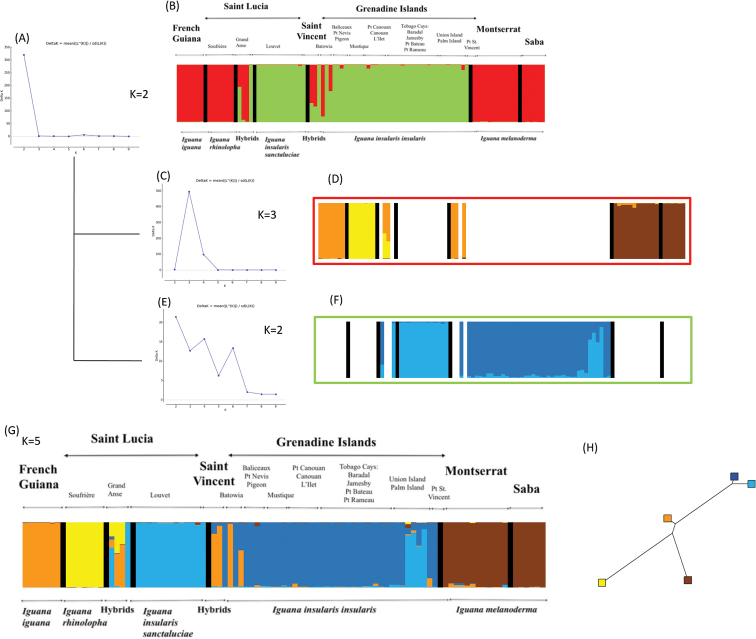
Hierarchical Structure analysis: **A** delta K method estimating that the uppermost hierarchical structure is composed of two main genetic clusters **B** corresponding barplot showing each individual as a vertical bar where each color corresponds to the admixture coefficient of the inferred genetic clusters; additional analyses within each of these two genetic clusters **C-F** showed that the first genetic cluster is composed of three genetic clusters (**C, D**) and the second one of two genetic clusters (**E, F**) totalizing an overall number of five genetic clusters across the whole dataset **G** genetic distance tree between genetic clusters based on the allele frequencies divergence among clusters **H**.

Results revealed significant genetic differentiation between groups of individuals (mean *Fst* value = 0.55) (Table 3). DAPC (Fig. 6) clearly suggested a strong genetic differentiation between five groups: alien individuals from St. Lucia (*I.rhinolopha*), native individuals from both Montserrat and Saba (endemic *I.melanoderma*), individuals from St. Lucia (endemic *I.insularissanctaluciae*), individuals from the Grenadines (endemic *I.insularisinsularis*), and French Guiana (*I.iguana*). It confirmed there is clear genetic differentiation between *I.insularisinsularis* from the Grenadines and *I.insularissanctaluciae* from St. Lucia. Moreover, the populations of St. Vincent and Grand Anse (St. Lucia) show mostly hybrids (*I.insularis* admixed with alleles from *I.iguana* and *I.rhinolopha*; Fig. 7).

**Table 3.** Comparison of *Fst* values for each pairwise group of individuals (below diagonal) and their significance (above diagonal). P-value threshold after Bonferroni adjustment, P = 0.0024. NS = not significant; * = significant. Mean *Fst* values: 0.55. The iguanas of St. Vincent were included in the group *insularis* according to their geographical origin but are hybrids as this was discovered by this work.

		1	2	3	4	5	6	7
**1**	*Iguanaiguana* French Guiana	–	NS	NS	*	*	*	NS
**2**	*I.rhinolopha* St Lucia	0.53	–	NS	NS	*	NS	NS
**3**	Hybrid St Lucia Grand Anse	0.26	0.45	–	NS	*	*	NS
**4**	*I.insularissanctaluciae* St Lucia Louvet	0.74	0.82	0.54	–	*	*	*
**5**	*I.insularisinsularis* St Vincent and the Grenadines	0.53	0.72	0.38	0.34	–	*	*
**6**	*I.melanoderma* Montserrat	0.38	0.63	0.45	0.81	0.64	–	*
**7**	*I.melanoderma* Saba	0.46	0.66	0.50	0.87	0.71	0.11	–

Moreover, the twenty independent runs implemented in the structure and structure harvester software revealed the highest mean likelihood for *K* = 2 genetic clusters (Fig. 7). Indeed, the main genetic structure we found clearly distinguished individuals of both *I.insularisinsularis* and *I.insularissanctaluciae* (from the Grenadines and St. Lucia, respectively) from other taxa (i.e., *I.rhinolopha*, *I.iguana*, *I.melanoderma*). The results also highlighted individuals showing intermediate admixture coefficients on both St. Lucia and St. Vincent, suggesting hybridization (Figs 5, 7). With *K* = 5, the STRUCTURE software also clearly separated genetic clusters that fit well with the five taxa (Figs 6, 7).

## ﻿Mitochondrial and Nuclear Genes

Of 14 individuals from St. Vincent (*n* = 3) and the Grenadine Islands (*n* = 11) sequenced for ND4 for this study, the haplotype of St. Lucia AF217782 identified by Stephen et al. (2013) was found on St. Vincent (*n* = 3), Battowia (*n* = 3), Tobago Cays (*n* = 3), Pigeon Island (*n* = 1), Union Island (*n* = 1) and Petit St. Vincent (*n* = 1). Moreover, an *insularis* haplotype (MK687402-3) previously identified by Breuil et al. (2019) on Palm Island was also found on Jamesby (MN590142) and Mustique (MN590150).

The ND4 haplotype AF217782 identified by Stephen et al (2013) in St. Lucia is also present in *I.insularisinsularis*: the three hybrids from St. Vincent, the three from Battowia, in three of four individuals from the Tobago Cays (Baradal, Petit Bateau, Petit Rameau), in the one from Petit St. Vincent, and in one of the two iguanas from Union Island. Palm Island has three different haplotypes, but we did not find AF217782 here. Jamesby (Tobago Cays) and Mustique have another haplotype. Thus of the 28 sequences available for *Iguanainsularis* (Stephen et al. 2013; Breuil et al. 2019, this study) none of the haplotypes of this species were observed among the 201 sequences studied (Stephen et al. 2013: *n* = 73; Breuil et al. 2019, 2020: *n* = 19; De Jésus Villanueva: *n* = 109). Breuil et al. (2019, 2020) published two median-joining haplotype networks for ND4 showing the independent position of these *insularis* haplotypes at the end of a branch.

All the sequenced iguanas from the Grenadines (*n* = 10) had the same endemic haplotype of the PAC gene (JN811117) as the one found in Louvet (St. Lucia) by Stephen et al. (2013) with *n* = 6. This haplotype differs by a G instead of a A at site 323. Moreover, we also found this haplotype in three hybrids from St. Vincent that were homozygous for this gene. Conversely, the population of Grand Anse (St. Lucia) had two iguanas (IGU53, IGU57) with this endemic JN811117 haplotype, whereas two others (IGU55, IGU56) are homozygous for a widespread Central American haplotype (JN811107).

The haplotype (JN811117) of the PAC gene identified by Stephen et al. (2013) in six individuals from St. Lucia was also found in all individuals of *I.insularis*: three from St. Lucia (Louvet), ten from the Grenadines and three hybrid individuals from St. Vincent. Two iguanas from the Grand Anse (St. Lucia) hybrid population out of the four sampled are homozygous for this haplotype while the other two recognized as hybrids (Breuil et al. 2019) are homozygous for a haplotype (JN811107) present in iguanas from Belize, El Salvador, Honduras, Guatemala, and Nicaragua. For example, the 70 PAC gene sequences obtained by Stephen et al. (2013), the 86 sequences from invasive exotic iguanas (De Jésus Villanueva et al. 2021) and the 19 from this study reveal 25 different haplotypes for this gene. Of 175 iguanas sequenced for this gene, haplotype JN811117, found nowhere else, unambiguously identifies *Iguanainsularis* among all other lineages and thus confirms the complex hybridization history of some populations.

## ﻿Discussion

### ﻿Distribution of *Iguanainsularis* on the St. Vincent and Grenada/Grenadines banks

All 34 iguanas sampled on 15 islands across the Grenadines during the present survey were identified as *I.insularisinsularis* and their morphology reinforced the assertion by Breuil et al. (2019) that the subspecies insularis and *sanctaluciae* can be reliably distinguished as adults from their coloration: Adult *I.insularisinsularis* are characterized by a beige, cream or even dirty white dewlap with relatively narrow black body stripes that tend to fade with age, whereas *I.insularissanctaluciae* typically develops a black dewlap and has relatively wide black body stripes that become even more pronounced with age.

Results from microsatellites from the 34 new samples from the Grenadines were found to be in accordance with morphological data. This study confirms the presence of *Iguanainsularisinsularis* on multiple Grenadine Islands based on genetic data. Both STRUCTURE and DAPC genetic analyses confirmed there is a clear differentiation between *I.insularisinsularis* and *I.insularissanctaluciae* (which was reported by Breuil et al. 2019 but based only on four genetic samples from the Grenadines). The *Fst* value was around 0.32, which supported a strong genetic difference between the two subspecies.

While *I.insularissanctaluciae* is restricted to St. Lucia (Breuil et al. 2019), the new genetic evidence from the Grenadines shows *I.insularisinsularis* extends from Bequia (North) to at least as far south as Petit St. Vincent. Gaymes and Justo-Gaymes (2018) observed iguanas with this phenotype on 19 of the 24 islands surveyed within the national boundary of St. Vincent and the Grenadines. Photographs of iguanas nesting in April 2020 on Anse la Roche, Carriacou, and iguanas in December 2020 on Mabouya Island, two of the Grenadine Islands in Grenada, were also consistent with *I.insularisinsularis* (Juliana Coffey in litt. to JD). Indeed, the native range of this taxon might extend as far south as the main island of Grenada, as suggested by the pioneering work of Lazell (1973), which shares the same geological bank as the Grenadines. Photographs of Grenadian iguanas published in Henderson and Powell (2018: 43, 267), however, show a combination of morphological characteristics that indicates the presence of hybrids with *I.iguana* and *I.rhinolopha*.

One of the challenges to elucidating the natural ranges of iguanas in this region is that they are often transported by people. For example, it is common practice for hunters to collect live iguanas from the Grenadines to sell as bushmeat on St. Vincent and Grenada during the hunting season from October through January (GG, pers. obs.). Daudin and de Silva (2011) reported that hunting is frequent on the uninhabited island of Baliceaux, from which hunters from Bequia and St. Vincent “carry away dozens of iguanas”. Some years ago, 30 iguanas from Palm Island were translocated to Petit St. Vincent, and in 2005 the St. Vincent & the Grenadines Forestry Department relocated 260 iguanas from Palm Island to the nearby Tobago Cays (also in the Grenadines) and to the Kingstown Botanical Garden on St. Vincent (Daudin and de Silva 2011). In 2020, 20 iguanas from Palm Island were translocated to Union Island by the Forestry Department, again in response to complaints from the Palm Island Resort. These translocations show the impact of human on the distribution and genetic structure of iguana populations in this region and thus it might be supposed that no population has remained untouched. Natural dispersal between nearby islands may also serve to homogenize these populations.

### ﻿Spread and impacts from alien iguanas: introgression of *Iguanainsularis*

Our samples from Battowia, Baliceaux, and Petit Canouan (Grenadine Islands) showed evidence of introgression of *I.insularisinsularis* by South American *I.iguana.* Similar observations were reported by Breuil et al. (2019) in one specimen of *I.insularisinsularis* on Union Island that harbored a South American mitochondrial haplotype (based on the analyze of the ND4 *loci*MK687401). Introgression is already a well-known phenomenon in iguana populations, notably between *I.iguana* and *I.delicatissima* in Guadeloupe and Anguilla and between *I.rhinolopha* and *I.delicatissima* in St. Barthélemy (Breuil 2013, 2016; Vuillaume et al. 2015; Pounder et al. 2020) and St. Eustatius (van den Burg et al. 2018). *Iguanarhinolopha* is an invasive alien species in the Lesser Antilles and is inferred to have originated from breeding farms in Central America (Costa Rica, Nicaragua, Honduras, Guatemala, El Salvador), which supply the pet trade (Stephen et al. 2011). *Iguanaiguana* is also invasive in the Lesser Antilles and may have arrived in St. Vincent and in the Grenadine Islands from the allochthonous *I.iguana* population of Martinique, where the species was introduced from Les Saintes and thus from French Guiana (Breuil 2009). In the light of both genetic and morphological results, the islands where *I.insularisinsularis* specimens showed no or very low introgression with continental alien iguanas were Baliceaux, Petit Nevis, Mustique, Pigeon (Ramier), Petit Canouan, L’Islet, and the Tobago Cays (Baradal, Jamesby, Petit Rameau, Petit Bateau). On Battowia, Union Island, Palm Island, and Petit St. Vincent, on the other hand, the situation is more complex with some individuals being admixed with continental iguana alleles and, on Union and Palm islands, *I.insularissanctaluciae* alleles.

While *I.insularisinsularis* is considered endemic to the Grenada Bank (which includes the Grenadines) and *I.insularissanctaluciae* is endemic to St. Lucia, Breuil et al. (2019) were unable to identify the taxon indigenous to the St. Vincent Bank, which lies between the two (Fig. 1B). The present study was unfortunately unable to answer this question definitively because all four iguanas sampled on St. Vincent during the present survey were found to belong to a hybrid population. All three that were photographed had a phenotype closely resembling French Guiana *I.iguana* whereas their PAC and ND4 sequences are *insularis* and their microsatellites are a combination of *Iguanaiguana*, *I.rhinolopha*, and *I.insularisinsularis*. We have no data on the morphology of the fourth specimen, IGU132, but its microsatellite genotype corresponded with *I.insularisinsularis*. It is, however, impossible to establish whether this last specimen represents an endemic iguana from the original population of St. Vincent or is the descendant of released or escaped iguanas from the Grenadines (e.g., Daudin and de Silva 2011). The ND4 haplotype of these three St. Vincent iguanas shows that the maternal lineage is *I.insularis* and this haplotype is the most common found in this species (MN590151-53). The homozygous PAC diagnostic locus (JN811117) shows that have been at least backcrosses with *I.insularis*. The microsatellites also indicate that this population is deeply introgressed. We see on St. Vincent an important discrepancy between the genetic analysis of this hybrid population and its morphology, which seems to indicate a nearly pure *Iguanaiguana* population.

We have not found any genetic or morphological signs of *I.delicatissima* in the iguanas from St. Vincent and the Grenadines. Conversely, in one population sampled on St. Lucia (Grand Anse, *n* = 4), Breuil et al. (2019) found two I.insularissanctaluciae with a delicatissima mitochondrial haplotype (MK687394-95). Moreover, these two individuals are homozygous for a PAC Central American haplotype (JN811107) and present microsatellites typical from *I.iguana* and *I.rhinolopha*. The third individual has a delicatissima ND4 like haplotype (MK687392) and is homozygous for the PAC *insularis* diagnostic haplotype (JN811117), while its microsatellites show introgression with both *I.iguana* and *I.rhinolopha*. The fourth individual from this population has ND4 and PAC haplotypes and microsatellites typical from *I.insularissanctaluciae*. Thus, this St. Lucian population demonstrates the complexity of hybridization in the genus *Iguana*, where some individuals possess genetic sequences belonging to four phylogenetic species that all have the ability to breed and produce fertile offspring, as demonstrated by the backcrosses. One of us (JD) was told in May 2021 that the Forestry Department of St. Lucia used to take iguanas handed in by the public to release at Grand Anse. This was done at a time where the morphological differences between iguanas were not well understood and these unfortunate translocations may well explain the alien genes in that population.

### ﻿Implications of these findings for the conservation of *Iguanainsularis*

As a result of both deliberate and accidental transportation, invasive alien iguanas (*I.iguana* and *I.rhinolopha*) and their fertile hybrids are now widely scattered across the Eastern Caribbean and pose a serious threat to all remaining indigenous populations of *I.insularis*, *I.melanoderma* and *I.delicatissima* (Breuil et al. 2019, 2020; Pounder et al. 2020; van den Burg et al. 2018; Breuil 2021). Indeed, a number of populations have already been lost following incursions by invasive alien *Iguana* species, e.g., *I.delicatissima* from most of Guadeloupe after the arrival of *I.iguana* (Vuillaume et al. 2015). The spread of alien lineages is likely to accelerate, with increasing traffic between islands and the projected increase in the frequency and severity of hurricanes due to climate change. Shortly after Hurricane Maria struck Dominica in September 2017, conservationists on the island discovered an incursion of alien iguanas that were inferred to have arrived on cargo boats with relief supplies, posing a major threat to the indigenous *I.delicatissima* population (van den Burg et al. 2020, 2021). At the time of writing, St. Vincent, reeling from a series of volcanic eruptions that began in April 2021, is receiving humanitarian aid on boats from Martinique and other islands whose harbors are infested with hybrid iguanas. Further arrivals of alien iguanas could eventually result in the progressive genetic absorption of *I.insularis*. The available evidence, mainly photographs, suggests that this may have already been the fate of the native iguanas on the main islands of St. Vincent and Grenada. Further surveys are urgently needed on both islands to determine whether any intact populations of their native iguanas remain.

These findings have important implications for conserving *I.insularis*. Most of the known populations are small, fragmented and exposed to multiple anthropogenic threats in addition to the alien iguanas. St. Lucia’s native population (subsp. sanctaluciae) is severely depleted and restricted to northeast St. Lucia, where it is under immense pressure from habitat loss, illegal poaching for bushmeat and the international pet trade, and feral and invasive alien mammals. Furthermore it faces a rising population of invasive alien *I.rhinolopha* that is spreading from southwest St. Lucia (Krauss et al. 2014). This subspecies clearly meets the IUCN criteria for Critically Endangered (Breuil et al. 2019), meaning it is at high risk of extinction. As shown in this paper, there is already clear evidence of hybridization in Grand Anse, an area that was erroneously believed to contain only purebred St. Lucia iguanas.

The situation looks somewhat brighter in the Grenadines, where the native iguanas (subsp. insularis) still occupy at least 21 of the 35 named islands. Unfortunately, most of these sites are very small and unprotected, and there is little to prevent incursions of alien iguanas from St. Vincent or Grenada, especially during the hunting season when live iguanas are openly transported between islands. Evidence of past hybridization with *I.iguana* was detected on several islands. Other threats observed during our field surveys included invasive alien cats, dogs, and rats (which prey on iguanas and eggs), domestic goats (which destroy vegetation), and bushfires (including the near-annual fires on Petit Canouan lit by seabird egg-collectors) (Gaymes and Justo-Gaymes 2018; Daltry and Steele 2020). Iguanas are hunted across the Grenadines for meat and, increasingly, for the international pet trade. A recent study found the “Grenadines pink rhino iguana” among the top three reptiles traded from the Eastern Caribbean (Noseworthy 2017). By recognizing the new species and two subspecies, we realize that the demand from reptile collectors could increase (Auliya et al. 2016) and this must be countered by increased protection both locally and internationally. We therefore uphold the recommendation in Breuil et al. (2019) to upgrade *Iguanainsularis* from CITES Appendix II to Appendix I. We also call for tighter controls on the movements of iguanas between islands, even within national borders, to avoid unsustainable hunting and reduce the spread of alien iguanas.

### ﻿Differentiation of *Iguanainsularis*

The microsatellites used in this study clearly show the uniqueness of the *insularis* lineage compared to other representative populations in the *Iguanaiguana* complex. Furthermore, comparison of the two subspecies of *insularis*, in a broader geographic context (van den Burg et al. 2021) including individuals from the different clades identified by Stephen et al. (2013), supports the originality of this species and its separation into two subspecies.

All of these genetic data (unique PAC and ND4 haplotypes) also confirm the uniqueness of the iguana populations of the southern Lesser Antilles, which were first identified by Lazell (1973). The combination of different morphological characteristics (scales, color) gives them a unique phenotype found nowhere else. These distinctive features have been acquired through an independent evolutionary history and are arguments for the recognition of the southern Lesser Antilles populations as a species with two easily identified subspecies which share morphological and genetic synapomorphies. However it would be similarly reasonable to hypothesize that *insularis* and *sanctaluciae* are subspecies of *Iguanaiguana* along with *melanoderma* and *rhinolopha*. Based on our data (morphology, genetic), we lean towards splitting this complex into several species, but we know well that further research is needed, especially in South America, to get a consensus for the taxonomy of this iconic lizard.

## ﻿Conclusion

The current range of the southern Antilles horned iguana *Iguanainsularis* includes St. Lucia (subsp. sanctaluciae) and at least 21 islands in the Grenadines (subsp. insularis). The first descriptions of these taxa were informed by genetic analysis of a relatively large number of individuals from St. Lucia but only four from the Grenadines (Breuil et al. 2019). The present paper adds genetic data from a further 34 individuals in the Grenadines and, for the first time, four from St. Vincent. Seventeen microsatellites, PAC and ND4 genes were used to estimate genetic diversity, population structure, and differentiation between the two subspecies as well as the level of introgression with other *Iguana* species. The results support recognition of *Iguanainsularis* as an independent lineage and also confirms there is a clear genetic differentiation between *I.insularisinsularis* and *I.insularissanctaluciae*. Because gene flow with introgression exists between all these five species, these recognized taxa do not fit the biological species concept and thus could be considered as subspecies of *Iguanaiguana* (as suggested by the Reptile Database, 2021). However, this gene flow is a recent phenomenon due to human activities. These anthropogenic movements of iguanas have disrupted the normal and independent evolution of these different island populations.

Despite having only recently been described, *Iguanainsularis* faces multiple threats, including unsustainable hunting, habitat loss and invasive alien species, including alien iguanas. While purebred *I.insularisinsularis* populations survive on several islands in the Grenadines, our results reveal evidence of hybridization with *I.iguana*, an invasive alien species from South America, and *I.rhinolopha* from Central America. The *I.insularis* population of St. Vincent shows a high level of introgression from *I.iguana*, while on St. Lucia, a growing population of invasive Central American *I.rhinolopha* endangers the remnant population of *I.insularissanctaluciae.* Experiences from other islands suggest that both invasive alien species are capable of driving native iguana to extinction through competition and introgression. Stronger protection of *I.insularis* is required throughout its range, coupled with concerted efforts to curb the spread of alien iguanas, *I.iguana* and *I.rhinolopha*.
